# Prevalence of Root Dilaceration in Adult Patients Referred to Shiraz Dental School (2005-2010)

**Published:** 2013-12

**Authors:** MR Nabavizadeh, M Sedigh Shamsi, F Moazami, A Abbaszadegan

**Affiliations:** a Dept. of Endodontics, School of Dentistry, Shiraz University of Medical Sciences, Shiraz, Iran

**Keywords:** Prevalence, Root Dilacerations, Periapical Radiography

## Abstract

**Statement of Problem: **Dilaceration is defined as a sudden change in the axial inclination of root or between the crown and the root of a tooth. There is no previous study evaluating its prevalence in south of Iran.

**Purpose: **This study evaluates the prevalence of root dilaceration on the basis of its location in dental arch in a sample of dental patients referring to Shiraz dental school, Iran.

**Materials and Method: **This retrospective study was performed using full mouth periapical radiographs of 250 patients who were referred to Shiraz dental school. Buccal and lingual dilaceration was determined by its known” bull’s eye” appearance in the radiographs or if the deviation was in the mesial or distal directions; the angle of 90 degree or greater between the deviation and the axis of root was the inclusion criteria.

**Results:** Root dilaceration was detected in 0.3% of teeth and 7.2% of patients. It was distributed equally between the maxilla and mandible. Mandibular second molar was the most frequent dilacerated tooth (1.6%) followed by maxillary first molar (1.3%) and mandibular first molar (0.6%). The alveolar nerve was the most common anatomic structure near dilacerated teeth.

**Conclusion:** According to this study, root dilaceration is an uncommon developmental anomaly which occurs mostly in the posterior teeth.

## Introduction

By definition, dilaceration is an abnormal angulation or bend in the root and less frequently, the crown of a tooth. Most cases are idiopathic and have no clinical feature [1]. In limited cases with recognized cause, injury was the first reason. Trauma can displace the calcified portion of tooth germ so that the non-calcified part of tooth is formed in an abnormal angle. In rare cases, this bending occurs due to the presence of cysts, tumors or hamartomas [2]. While it may be clinically detected by palpation high in the labial sulcus or hard palate [3]; periapical radiography is the best method to detect this abnormal condition and is characteristic [3-4]. The mesial or distal dilaceration is obviously detectable in periapical radiographs but buccal or lingual dilaceration appears as a round opaque region with radiolucent area in its center (bull’s eye appearance) [1]. The process of endodontic treatment in its all stages including diagnosis, access cavity preparation, cleaning and shaping and obturation might be difficult in these cases [4]. So its diagnosis and awareness of its prevalence are important for endodontic treatment and any overlook may cause higher rate of failure of endodontic treatment in these teeth [5]. Studies, evaluating its prevalence in different races; have revealed different results [6-10]. Therefore, studying its racial prevalence seems to be relevant and justified. There is no study evaluating dilaceration in the Fars province population since publishing this article. The aim of this study is to determine the prevalence of dilacerations in adult population of this region of Iran.

## Materials and Method

**Figure 1:**
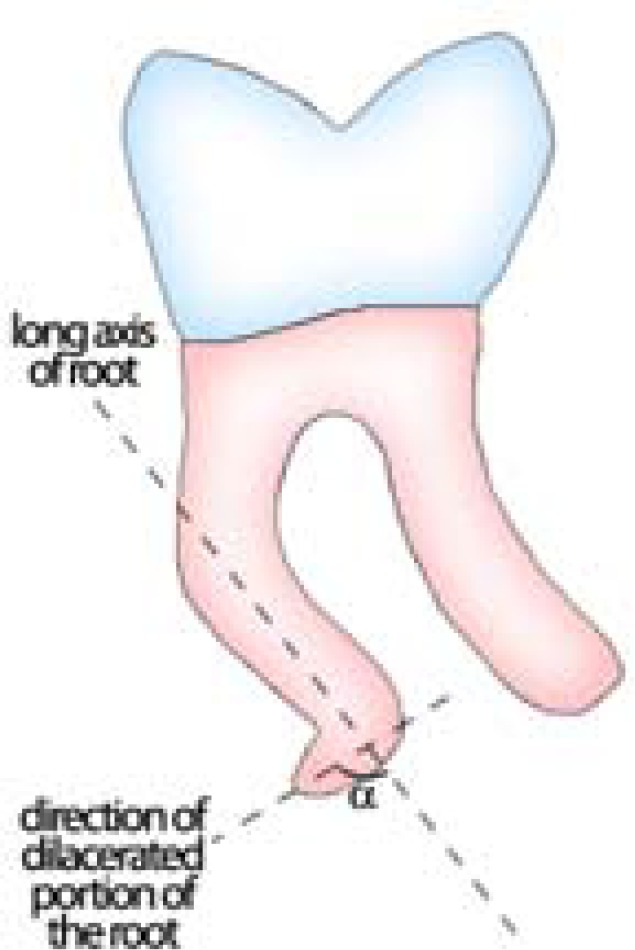
Root dilacerations in mesiodistal plane was noted if the angle between the long axis of the root and the axis of dilacerated portion of the root (α) was >90

This descriptive study was enrolled by taking a random sample of 250 records out of 1071 dental records registered from years 2005 to 2010. These records contained full mouth periapical radiographs from patient who attended endodontic and periodontal departments of dental school of Shiraz University of Medical Science. The radiographs have been taken for endodontic and periodontal diagnostic purposes. The patients were 12 to 75 years old and all patients were Caucasians who were living in Iran. The radiographs were taken by parallel technique in oral and maxillofacial department of Shiraz Dental School. Poor-processed radiographs, radiographs taken with improper angles, blurred images and repeated radiographs from same region were exclu-ded from the study to reduce the possible misinterpretation. The ultimate selection that included 2231 periapical radiographs with 6146 teeth were evaluated for the presence of dilacerations by two endodontists separately with magnifying lens (3×) and X-ray viewer. When bull’s eye appearance (round opaque area with radiolucency in its central region) in the radiographic image of root(s) of a tooth was detected, it was categorized as the buccal or lingual dilaceration. If root deviation oriented mesialy or distally, the direction of dilacerated portion of the root and long axis of tooth were drawn on the orthodon-tic tracing paper. The angle of 90^о ^or more was consider-ed as a dilaceration (Figure 1). The deviation was categ-orized to either apical, middle, or the coronal third of the root.

When there was no agreement between two examiners, they revised the radiograph together to reach to a similar decision. If a multirooted tooth had dilaceration in one of its roots, it was considered as a case of dilaceration. The proximity of each dilacerated tooth to the anatomic regions including nasal fossa, maxillary sinus, mental foramen and alveolar nerve was assessed. Finally, the percentage of dilacerations of each root was expressed as the descriptive statistic results. SPSS (v18) software (SPSS; Chicago, IL, USA) was adopted to analyze the obtained data. The incidence of dilaceration in two jaws was compared by chi-square test and the critical level of significance was 0.05.

## Results

**Figure 2a:**
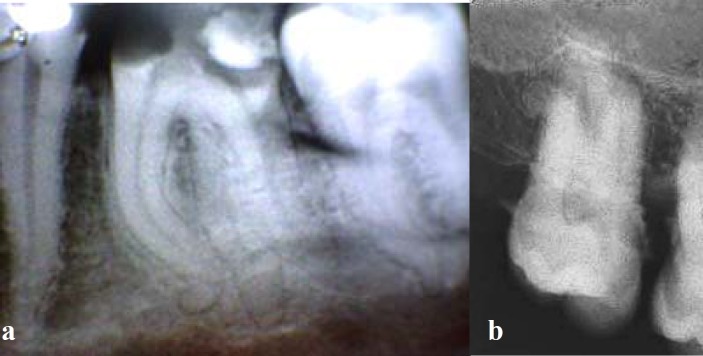
Root dilaceration in mesial root of a first lower molar **b** Root dilaceration in distobuccal root of a second upper molar

A total of 19 out of 6146 teeth were diagnosed as being dilacerated teeth (0.3%) (Figure2). A total of 18 patients out of 250 patients, (7.2%) had at least root dilaceration in one of their teeth.

**Figure 3:**
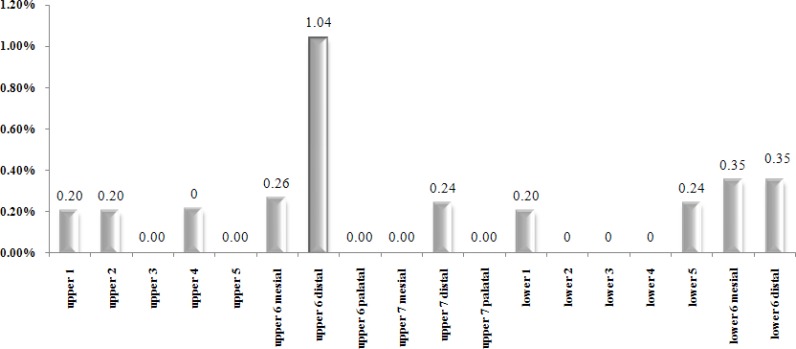
Percentage of dilaceration in each root

Root dilaceration was similarly distributed in two jaws (*p*= 0.768>0.05).Mandibular second molar was the most frequent dilacerated tooth (1.6%) followed by maxillary first molar (1.3%) and mandibular first molar (0.7%). Dilaceration was not detected in the maxillary canine, second premolar and mandibular lateral incisor, canine and first premolar. Regarding individual root dilaceration, it was detected mostly in distal root of mandibular second molar (1.06%), distal root of upper first molar (1.04%) and mesial root of lower second molar (0.53%) (Figure 3). Only one dilacerated first upper premolar was near maxillary sinus and one out of the four dilacerated first upper molars was near maxillary sinus while four out of the six lower second molars were adjacent to the alveolar nerve (Table 1). 

## Discussion

The study showed that dilacerations were more prevalent in the posterior teeth. For a precise diagnosis of root dilaceration, radiological examination is almost compulsory [4]. Panoramic radiography is not a proper choice to detect root dilaceration due to its lower accuracy [11] and its limitation to identify buccal and lingual dilacerations. Periapical radiography is the best choice for this purpose [4]. Malcić’s et al. reported that the periapical radiographs are more sensitive for detecting dilacerated maxillary central incisors [8]. So in this study, we used full mouth periapical radiographs which were already taken for endodontic and periodo-ntal evaluations. There are various dissimilar definitions for dilacerations in mesial and distal direction.

**Table 1:** Total number of examined teeth; dilacerated teeth and their vicinity to the anatomic regions

**Tooth**	**Number of** **examined teeth**	**Number of total** **Dilacerations (%)**			**Apical** **third**	**Middle** **third**	**Number of teeth** **near anatomic regions**	**Adjacent anatomic** **region**
**Maxillary**	**3121**	9(0.28%)			7	2		
Central incisor	486	1(0.2%)			1	-	-	-
Lateral incisor	485	1(0.2%)			1	-	-	-
Canine	465	0(0%)			-	-	-	-
First premolar	465	1(0.2%)			1	-	-	Maxillary sinus
Second premolar	440	0(0%)			-	-	-	-
First molar	366	5(1.3%)	Mb*:1		3	2	1	Maxillary sinus
			Db†:4					
Second molar	414	1(0.2%)	Db:1		1		-	-
**mandibular**	3025	10(0.33%)			7	3		
Central incisor	491	1(0.2%)			1			
Lateral incisor	494	0(0%)			-	-	-	-
Canine	499	0(0%)			-	-	-	-
First premolar	474	0(0%)			-	-	-	-
Second premolar	415	1(0.2%)			1	-	-	-
First molar	278	2(0.7%)	M‡:1		1	1	-	-
			D¶:1					
Second molar	374	6(1.6%)	M:1		4	2	4	Alveolar nerve
			D:5					

* Mesiobuccal root dilacerated in a multirooted tooth.                   † Distobuccal root dilacerated in a multirooted tooth.

‡ Mesial roots dilacerated in a multirooted tooth.                    ¶ Distal roots dilacerated in a multirooted tooth.

Some authors define dilaceration as a deviation of 90 degree or greater from the normal axis of the tooth [8-9]. Others considered a tooth or a root to have dilaceration if there was 20 degree or more deviation from the normal axis of the tooth [5]. In this study; samples were classified based on the first definition due to its higher accuracy.

Our study depicted a 0.3% dilaceration which is much lower than findings of Milogu et al. [6] in Turkey (9.5%), Udoye et al. [7] in Nigeria (3%), Hamasha et al. [9] in Jordan (3.8%) and Ezoddiny et al. [12] in Yazd (15% of patients). Our results are near to Malcić’s et al. findings that reported incidence of 0.32% for dilaceration in Croatia [8]. The difference in diagnostic criteria might be the cause of this dissimilarity.

According to our results, there was no significant difference between two jaws in the prevalence of dilaceration. These results are consistent with Milogu’s et al. [6] findings but not with Malcić’s et al. [7] or Hamasha’s et al. [9] results which expressed higher distribution in maxilla than mandible.

There is a variety of suggestions concerning the dilaceration etiology. The oldest and the most proposed etiology for dilaceration is trauma to deciduous tooth when calcified segmentof the underlying permanent tooth germ is forming [7, 12-13]. Most of the traumatic injuries occur in children with the mean age of 4 years old. At this age, up to half of the crown is developed [14]. Andreasen et al. believe that three percent of trau-matic injuries to deciduous teeth ends up to this type of anomaly, specially avulsion and intrusion injuries [14]. 

This theory is confirmed with the studies that reported higher percentage of dilaceration in anterior teeth [13-14]. However, many studies show higher perc-entage of dilaceration in molar and premolar teeth that their tooth germ is not near any deciduous tooth or trauma to their corresponding deciduous teeth is rare [1, 6-9]. We also found higher incidence of the anomaly in posterior teeth which propose idiopathic developmental disturbance in tooth germ calcification as a possible cause.

Other mentioned contributory factors include pre-vention of normal eruption of permanent teeth by scar formation caused by trauma to the nearby deciduous teeth, the primary tooth germ anomaly, and also [15] deflection of epithelial diaphragm by anatomic structu-res such as cortical bone of maxillary sinus, mandibular canal and nasal fossa [16] or pathologic lesions such as dentigerous cyst [17] and compound odontoma [18]. The detected dilacerated teeth in this study were neither near any pathological lesions nor the nasal fossa. Only one of the five dilacerated first molars was near maxillary sinus. Also the only one first upper premolar with dilacerated root was near this anatomic region. Most of the second mandibular molars (four of six) had proximity to the inferior alveolar nerve as an anatomic region that may change the direction of root during its development.

Some studies reported higher incidence of this dental anomaly in patients suffering from specific syndromes like Ehlers-Danlos syndrome [19] or Smith-Magenis syndrome [20]. However; we did not find any special systemic complication in the records of the patients recruited in our study.

Exploration and negotiation of root canal system is difficult in dilacerated teeth due to its high degree of curvature. The rate of endodontic errors such as ledging, transportation and zipping can be higher in these teeth [4]. Therefore, complete debridement of canals, elimina-ting microorganisms from it and its obturation becomes difficult. Knowledge of dilaceration prevalence and early diagnosis can help dentists to prevent these errors and improve the success rate by referring these cases to the specialists. Unwanted resorption of the root might happen if orthodontic forces are introduced to these teeth [3]. In case of extraction of these teeth, horizontal or vertical bone loss may also occur [3]. According to our results, practitioners must consider possibility of occurrence of this anomaly when taking treatment decision for the posterior teeth.

Cone beam computed tomography (CBCT) is a new imaging technique that can determine root canal curvature more precisely [21]. Therefore; it can be used for more accurate detection of the dilacerations in the future epidemiologic studies.

## Conclusion

Dilaceration is seen mostly in posterior maxillary and mandibular teeth regions. More accurate studies with employing new diagnostic radiographic techniques are needed for better assessment of this anomaly.
